# Operations upon Hemorrhoids*Diseases of the Rectum, London, 1871.

**Published:** 1872-06

**Authors:** William Allingham

**Affiliations:** Surgeon to St. Mark’s Hospital for Fistula


### OPERATIONS UPON HEMORRHOIDS.*

BY WILLIAM ALLINGHAM, F. R. S. C., SURGEON TO ST. MARK'S HOSPITAL FOR FISTULA.

The author of this excellent and most valuable work, in speaking of the relative merits of the ligature and the clamp cautery, gives the preference to the ligature, for which he claims the following advantages :

1st. The rapidity with which it may be executed. He has often operated upon four or five hemorrhoids, returned them, and removed redundant skin, in one minute and a half or two minutes.

2d. There is only a very small amount of tissue included in the ligature—in fact, little more than the vessels supplying the tumor.

3d. At least three-quarters of the wound is a simple incised wound, which heals rapidly, only the small portion included in the ligature having to slough away.

4th. The ligatures are tied a considerable distance from the anus, so that, when returned into the bowel, they lie above the internal sphincter, where the sensibility of the mucous membrane is not acute, and consequently the pain and irritation, after the operation, are reduced to a minimum.

5th. The operation is wonderfully free from danger to life, and its results are almost always satisfactory.

Mr. Allingham does not think that in the whole range of surgery there is any procedure worthy of the name operation which can show a greater amount of success.

In describing the mode of treating hemorrhoids and prolapsus by the ligature, Mr. Allingham refers to the operation about to be described, and not to the usual method of applying the ligature by transfixion of the base of the pile, and tying it in halves.

"This is the way in which the operation should be performed : I will premise, that in all operations about the rectum, but more particularly in cases of piles, it is essential that the alimentary canal should be thoroughly cleared before operating. The patient being thus prepared, administer an enema of warm water, and direct him to strain the piles well down while evacuating the injection; this being accomplished, place the patient upon a hard couch or mattress in a good light; the position should be on the right side, with a little tendency to roll over on to the stomach, the knees are to be drawn up to the abdomen, and if the patient

* Diseases of the Rectum, London, 1871.

has the courage to strain down during the operation, he will very much facilitate the manipulations. The assistant should stand with his back toward the patient's head, and raise the upper buttock with his right hand; at the same time, by hooking the elbow over the pelvis, he can keep the patient in position. The surgeon then, with a vulsellum or pronged hook, seizes the hemorrhoids, one after another, draws them down, and then, with a pair of sharp, strong spring-scissors separates the pile from its connec. tion with the muscular and submucous tissues upon which it rests; the cut is to be made in the sulcus or white mark which is seen where the skin meets the mucous membrane, and this incision is to be carried up the bowel, and parallel to it, to such a distance that the pile is left connected by an isthmus of vessels and mucous membrane only.

"There is no danger in making this incision, because all the large vessels come from above, running parallel with the bowel, *first beneath* the *mucous membrane*, and thus enter the *upper part* of the pile. A well-waxed, strong silk ligature is now to be placed at the bottom of the deep groove you have made, and the assistant then drawing out the pile with some decision, the ligature is tied high up at the neck of the tumor, as tight as possible. If this be done, *all the vessels must* be included. The silk should be so strong that you cannot break it by fair pulling. A portion of the pile may now be cut off, taking care to leave sufficient stump beyond the ligature to guard against its slipping. When all the hemorrhoids are thus tied, they should be *returned thoroughly* within the sphincter; after this is done, any superabundant skin which remains apparent may be cut off; but this should not be too freely excised, for fear of contraction on the healing. An injection of liq. opii sedativus may be administered. I always place a pad of wool over the anus, and a tight T-bandage, as it relieves pain most materially.

"After the operation, the bowels should be confined for three or four days by an astringent draught, such as: pulv. cret. aromat. scr. j; tinct. opii ♏xv; spt. æth. nit. dhr. j; mist. camphoræ, ad an oz. jss. To be taken night and morning, or three times in the day, for two days. This will very much assuage pain, prevent the tendency to strain, and keep the bowels confined. The diet at first, should be light, no alcohol at all should be taken; perfect rest in the recumbent position should be enjoined. On the third or fourth night a mild aperient may be administered, and after it has acted, a more liberal diet may be allowed."

Mr. Allingham thinks it advisable that the patient should keep lying down until the ligatures separate, which almost invariably takes place about the sixth or seventh day. *Active exertion*, even after the separation of the ligatures, is to be deprecated until the sores left in the rectum are quite healed; a fortnight is generally about the time required to accomplish this.

